# Local Binary Pattern–Cycle Generative Adversarial Network Transfer: Transforming Image Style from Day to Night

**DOI:** 10.3390/jimaging11040108

**Published:** 2025-03-31

**Authors:** Abeer Almohamade, Salma Kammoun, Fawaz Alsolami

**Affiliations:** 1 Department of Computer Science, Faculty of Computing and Information Technology, King Abdulaziz University, Jeddah 21589, Saudi Arabia; smohamad1@kau.edu.sa (S.K.); falsolami1@kau.edu.sa (F.A.); 2 Applied College, Taibah University, Almadinah Almunawwarah 41911, Saudi Arabia

**Keywords:** cycle generative adversarial network (CycleGAN), Local Binary Pattern (LBP), transform image style, unpaired image-to-image translation

## Abstract

Transforming images from day style to night style is crucial for enhancing perception in autonomous driving and smart surveillance. However, existing CycleGAN-based approaches struggle with texture loss, structural inconsistencies, and high computational costs. In our attempt to overcome these challenges, we produced LBP-CycleGAN, a new modification of CycleGAN that benefits from the advantages of a Local Binary Pattern (LBP) that extracts details of texture, unlike traditional CycleGAN, which relies heavily on color transformations. Our model leverages LBP-based single-channel inputs, ensuring sharper, more consistent night-time textures. We evaluated three model variations: (1) LBP-CycleGAN with a self-attention mechanism in both the generator and discriminator, (2) LBP-CycleGAN with a self-attention mechanism in the discriminator only, and (3) LBP-CycleGAN without a self-attention mechanism. Our results demonstrate that the LBP-CycleGAN model without self-attention outperformed the other models, achieving a superior texture quality while significantly reducing the training time and computational overhead. This work opens up new possibilities for efficient, high-fidelity night-time image translation in real-world applications, including autonomous driving and low-light vision systems.

## 1. Introduction

Autonomous driving systems use a strong vision model that can recognize road scenes. However, these systems encounter issues under low-light conditions, such as night-time, because of the lack of night-time images on which the deep learning algorithm can be trained. Unpaired image-to-image translation techniques, such as the traditional CycleGAN approach presented by Zhu et al. [1], can generate night-time images styled from daytime images and then use them in autonomous driving systems to enhance the robustness of the perception of these systems at night.

In this study, we introduce LBP-CycleGAN, a novel addition to traditional CycleGAN [1] that integrates Local Binary Patterns (LBPs) to improve texture preservation in day-to-night translation. Unlike conventional image translation approaches, which often focus on color transformation, our model emphasizes structural and textural consistency, ensuring that critical elements such as road markings, lane boundaries, and vehicle silhouettes remain intact in the translated night-time images.

CycleGAN [1] models are based on unpaired datasets, where images in the source domain (daytime) and target domain (night-time) do not have direct one-to-one correspondences. CycleGAN [1] learned in bidirectional mapping between a source and a target is not the same as in paired datasets, where the generated image is compared with the paired dataset. CycleGAN [1] learns by using two generators and two discriminators. The first generator, G(X), translates the image style from day to night. The second generator F(y), transforms the image style from night to day; in other words, it back-generates the image into the original style to enforce cycle consistency. This ensures that the translated night-time images remain faithful to the real-world distribution while avoiding structural distortions. Furthermore, by leveraging LBP-based single-channel inputs, our model reduces computational complexity, enabling faster training while maintaining high-fidelity night-time transformations.

Recent advances in image-to-image translation have introduced diffusion models as a powerful alternative to GAN-based architectures, achieving state-of-the-art results in various generative tasks. Diffusion models, such as DDPMs (Denoising Diffusion Probabilistic Models) and Latent Diffusion Models (LDMs), generate images by progressively refining noise over multiple timesteps, leading to high-quality, globally consistent outputs [2,3]. However, these models are computationally expensive, requiring hundreds to thousands of iterative steps for a single image transformation, making them impractical for real-time applications such as autonomous driving. In contrast, our LBP-CycleGAN operates in a single forward pass, significantly reducing inference time while preserving fine-grained texture details crucial for night-time road scene understanding. By leveraging LBP-based preprocessing, our method enhances structural clarity with minimal computational overhead, making it a viable alternative to computationally demanding diffusion models.

### 1.1. Key Contributions

Our research makes the following novel contributions:

Integration of LBP-Based Single-Channel Inputs: Unlike traditional CycleGAN, which operates on standard RGB images, our approach leverages LBP features as input to enhance texture preservation. This helps capture fine-grained details crucial for night-time transformations while reducing redundant color information.

Computational Efficiency: By using LBP images as input, our model reduces computational overhead, enabling faster training without sacrificing visual quality. This makes it more suitable for real-time applications, such as autonomous driving in low-light conditions. Using LBP images reduces the training time by approximately 2 days compared to traditional CycleGAN.

Analysis of Self-Attention in CycleGAN: We systematically evaluate the impact of self-attention in both the generator and discriminator, revealing that while self-attention enhances feature diversity, it does not always improve structural fidelity. Our results show that adding self-attention reduces the FID score by 34% compared to traditional CycleGAN, indicating a decrease in the quality of the image.

Optimized Model Variants: We propose and compare multiple LBP-CycleGAN architectures, demonstrating that removing self-attention can lead to better structural accuracy. In fact, removing self-attention entirely improves the FID score by 41.63% compared to using self-attention in both the generator and discriminator and by 8.00% compared to using self-attention in the discriminator only. This highlights that self-attention mechanisms may introduce unnecessary complexity without always benefiting image translation quality.

### 1.2. Organization of the Paper

The remainder of this paper is structured as follows. Section 2 provides a review of GAN-based image translation and its applications in general. Section 3 details our proposed LBP-CycleGAN architecture, including its network design, loss functions, and training strategy. Section 4 describes the experimental setup, datasets, and evaluation metrics, comparing the results from LBP-CycleGAN with baseline models, including an ablation study on self-attention mechanisms. Finally, Section 5 concludes the paper and outlines future research directions.

## 2. Literature Review

CycleGAN, or the cycle-consistent Generative Adversarial Network [1], was first introduced by Zhu et al. It is a type of generative adversarial network designed for image-to-image translation tasks without requiring paired training data. It was introduced to address the challenge of transferring styles between two domains (e.g., converting images from one artistic style to another) while preserving the underlying content.

The key innovation of CycleGAN is its use of cycle consistency loss. This means that if an image from domain A is transformed into domain B and then back into domain A, it should closely resemble the original image. This principle helps to ensure that important features are retained during transformation and prevents arbitrary changes that could misshape content.

CycleGAN consists of two main components: two generators and two discriminators. The first generator translates images from domain A to B (G), while the second generator performs the reverse (F). Each discriminator evaluates how realistic each generated output looks compared to dataset images in their respective domains.

Both groups of generators work with their corresponding discriminators when training through adversarial losses—by working against one another—and through cycle consistency losses, which enforce fidelity between input–output pairs under transformations. Thus, CycleGAN can learn successful mappings between unpaired datasets and can generate high-quality outputs that are suitable for many applications, e.g., style transfer or photo enhancement.

The most common reason for the use of CycleGAN is to augment rare datasets in applications where other methods have limited results. For example, a study [4] described the limitations in driving control caused by different road surface conditions and utilized CycleGAN to augment dry surface conditions to account for wet or snowy road surfaces. Further, in [5], the authors describe how bad weather or muddy roads can lead to the car’s fisheye camera becoming dirty, resulting in parts of the images being lost and affecting the car’s ability to perform certain autonomous driving activities, such as automatic parking. Other augmentation datasets focus on specific scenes in the road environment, such as pedestrians [6], in order to improve the ability of autonomous driving systems to detect pedestrians in certain conditions.

CycleGAN has been successfully used in the following applications:

- Photorealistic style transfer (e.g., summer ↔ winter) (such as in [7,8,9]).
- Artistic style transfer (e.g., photo ↔ Van Gogh) (such as in [10,11,12]).
- Medical imaging (e.g., CT ↔ MRI) (such as in [13,14,15]).
- Autonomous driving (e.g., day ↔ night, clear ↔ foggy) (such as in [16]). In this application, the system is designed to convert images taken during the day into images that look like they were taken at night, improving how autonomous driving systems perceive their surroundings in low-visibility conditions, such as at night or during bad weather. The network uses advanced techniques to understand both the meaning of objects in images (semantic information) and their shapes or structures (geometric information) to create more accurate night-time images. These networks are only trained on daytime images, which causes inaccurate feature extraction for night-time scenes. This limitation may reduce the model’s performance when applied to unseen night-time environments. The authors acknowledge the need for a domain-adaptive segmentation model capable of extracting meaningful features in both daytime and night-time conditions. The researchers in [17] used CycleGAN training, which combines different image processing techniques such as blurring and single-scale luminance transform (SLAT) processing, to improve the details and colors of the images while transitioning from day to night to create night-time images, which are then paired with real daytime images to form a dataset that focuses on converting daytime images into night-time images while preserving important details that help understand the scene. The researchers in [18] use a technique called transfer learning, where they first train a model on daytime images and then adapt it to generate realistic night-time images, ensuring that the essential information remains intact for better performance in driving simulations.

All of these applications are effective for unpaired image-to-image translation, but CycleGAN has several limitations that impact the quality and realism of generated images. One major limitation is its inability to handle long-range dependencies, as traditional convolutional layers have a limited receptive field, making it challenging to model global structures and relationships between distant regions. Another limitation of CycleGAN is its poor preservation of fine-grained details, particularly in high-resolution images, as the model often overlooks local textures and patterns.

## 3. Methods

### 3.1. Local Binary Patterns

**Figure 1:**
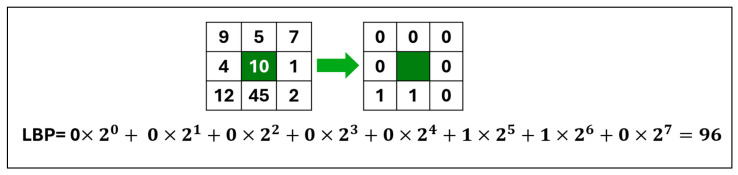
Example of LBP process.

Local Binary Patterns (LBPs) are a powerful texture descriptor used in image processing and computer vision. Ojala et al. [19] obtained the local texture patterns of an image by dividing it into blocks; each block measures 3 × 3 pixels and uses a center pixel as a threshold value for eight adjacent pixels. The center decimal value is calculated using the code from adjacent numbers with the following formula:

$$LBP=\sum_{i=0}^{p-1}s(n_{i}-G_{c}{)}^{2^{i}}$$

$$S\left(x\right)=\left\{\begin{array}{ll} \;1, & if\;x>0\\ 0, & otherwise \end{array}\right.$$

where *c* is the center pixel, $n_{i}$ denotes the ith surrounding pixel, p is the total number of neighborhood pixels, *Gc* is the decimal value, and s(x) is the sign function. The features for a histogram of size 2P are retrieved from the resulting LBP code. Thus, a histogram feature vector length of 256 is obtained for the eight adjacent pixels. In Figure 1, the LBP process is displayed with eight neighboring pixels and a Gc decimal value.

One of the key advantages of the Local Binary Patterns (LBP) approach is its inherent robustness to noise, which is particularly beneficial when working with grayscale transformations in low-light conditions. As described in the original LBP paper [19], LBP encodes texture patterns by comparing each pixel’s intensity value to that of its neighboring pixels. This process is less sensitive to small changes in pixel intensity due to noise, as minor intensity variations do not significantly alter the binary code.

Aside from this simple version of LBP, a number of extensions have been suggested throughout the years—such as Circular LBP—that consider different neighborhood configurations or utilize weighting schemes that place more emphasis on some features.

LBP is useful in several ways: it is a good representation of local patterns while being robust to monotonic gray-scale transformations, it is computationally lightweight, and it is easy to integrate into other algorithms such as classifiers or neural networks for tasks like face recognition and object detection and now increasingly within generative models such as GANs for tasks like style transfer. In the context of our LBP-CycleGAN model, the LBP constraints help ensure that texture features are preserved while minimizing the influence of noise, especially in night-time image translation, where noise can be more prominent due to low light levels.

Finally, Local Binary Patterns are not only useful as texture analysis tools but also as tools for enhancing visual quality by inserting them into more complex machine learning models.

In [20], the integration of LBPs enhances the model’s performance by providing more discriminative and robust features related to facial expressions. LBPs capture essential texture information, such as micro-expressions, which are crucial for distinguishing emotional states. By using these texture-based features as inputs, the CycleGAN model benefits from improved training data quality, enabling better generalization across different facial images. This pairing eventually results in better accuracy in identifying and categorizing children’s facial expressions since the LBP maintains essential local patterns, while CycleGAN allows for the conversion and learning of these patterns throughout the training process. In [21], the Circular Local Binary Pattern serves as a texture prior that assists in enhancing the fine textures of the produced style images. Adding LBPs to the GAN generator improves local texture feature extraction, resulting in higher quality and clearer details in output images obtained using style transfer. A prominent study proposed Circular LBP as a texture prior in a GAN model to enhance high-frequency details in artistic style transfer. The model aims to improve texture representation in the generator to generate visually pleasing stylized images. However, this approach primarily targets artistic applications rather than real-world image-to-image translation. In contrast, our proposed LBP-CycleGAN integrates the LBP as an input feature, leveraging its texture-preserving properties to improve realistic night-time image synthesis for autonomous driving applications. Unlike Circular LBP GAN, which emphasizes style adaptation, our model prioritizes structural integrity and computational efficiency, ensuring that translated night-time images retain critical features such as road markings, vehicle edges, and environmental details.

The LBP is not the only feature used in Generative models. For example, Mukherkjee et al. [22] introduced an aggregation-based GAN approach for generating synthetic brain tumor images, leveraging multiple GAN models with style transfer. The Sobel operator is applied after selecting the top two images generated by different GAN models based on similarity scores compared to the original images. The Sobel filter is used to detect edges by calculating the gradient of image intensity, producing edge-mapped versions of the selected images that emphasize their structural features. Following this, the Gaussian values of the intensities from these edge-mapped images are calculated to help combine the information from both images effectively. This aggregation method enhances the quality of the final image by balancing the edge details and smooth intensity distribution, leveraging the strengths of both GAN-generated images. Their work demonstrates that combining multiple generators improves fine-grained textural details in medical imaging. Unlike GAN aggregation, our approach modifies CycleGAN to incorporate LBP, ensuring improved texture consistency while maintaining structural integrity in transformed images. While alternative feature extraction techniques, such as Sobel filters [23], have been widely used for edge detection, they primarily focus on detecting gradient-based edge transitions rather than capturing local texture variations. Sobel operators are particularly effective for high-contrast edge detection in structured environments but tend to lose important textural details in complex real-world scenarios [24]. In contrast, LBP offers a more robust texture descriptor, encoding local structural patterns at the pixel level, making it highly effective for night-time vision tasks where preserving texture consistency is more critical than edge sharpness.

### 3.2. Self-Attention

Self-attention [25] is a mechanism produced by Zhang et al. that allows a model to weigh the importance of different parts of an input sequence when processing it. Unlike traditional attention mechanisms, which focus on aligning inputs with specific outputs, self-attention computes relationships within the same input data. This enables the model to capture long-range dependencies and contextual information effectively. In self-attention, each element in an input sequence can attend to all other elements, allowing for dynamic weighting based on their relevance. The process involves three main components: queries (Q), keys (K), and values (V). Each element generates these vectors through learned linear transformations. The attention scores are computed by taking the dot product between queries and keys, followed by applying a softmax function to obtain normalized weights. These weights are then used to create a weighted sum of value vectors. This approach has been particularly successful in natural language processing tasks but has also found applications in computer vision models. By applying self-attention modules in both the generator and discriminator, ESA-CycleGAN [26] can focus on different parts of the image more effectively. This allows the model to better understand the relationships between distant pixels and maintain important details during style transfer, ultimately leading to improved visual quality in the output images. The researchers in [27] integrate self-attention mechanisms into a CycleGAN framework, providing global context awareness, improving detail recovery, and emphasizing critical features such as fog density and scene details. Self-attention enables the model to capture long-range dependencies, preserve depth cues, and adapt its focus dynamically during training. This leads to better generalization across varying fog patterns, improved texture preservation, and fewer artifacts near object boundaries, ultimately resulting in clearer and more accurate defogged images. There are many types of attention mechanisms, such as self-attention, which selectively focus on important features to improve representation learning. For example, the researchers in [22] explore Latent Graph Attention (LGA) mechanisms to enhance feature propagation in CNN-based models, particularly for tasks such as segmentation and optical flow estimation.

While their study focuses on structured spatial learning in CNNs, it aligns with our investigation of self-attention mechanisms in CycleGAN for unpaired image translation. Our results indicate that self-attention does not always lead to improved performance in generative models, contrasting with their findings in feature learning for CNN-based tasks. This highlights the need for the task-specific optimization of attention mechanisms, as their effectiveness varies based on the architectural design and application domain.

### 3.3. CycleGAN

**Figure 2:**
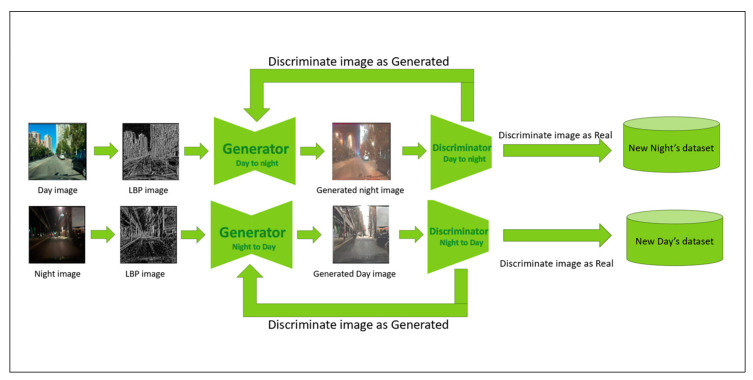
Architecture for proposed CycleGAN.

We considered various model architectures and aimed to provide the best model for transforming daytime images into night-time images. Our method involves modifying CycleGAN to overcome the challenges of poor preservation of fine-grained details by using an LBP image as input for the generator. Figure 2 illustrates the general architecture for LBP-CycleGAN, with two generators and two discriminators. The first generator generates images from day to night, and the discriminator determines whether they are real or generated. If the discriminator decides that the image is generated, the generated image is returned to the generator, which generates another enhanced night-time image. This process continues until a night-time image is generated that can fool the discriminator into recognizing it as a real image; then, the second generator and discriminator conduct the same process from night to day.

**Figure 3:**
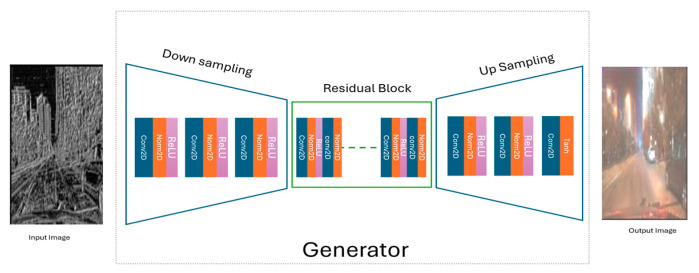
The generator in the LBP-CycleGAN.

Figure 3 illustrates the generator in our LBP-CycleGAN model. First, at the preprocessing input image level, we extract the LBP features from dataset images. In this stage, we input the image into the generator (LBP image).

The daytime LBP input image is processed by the generator, which consists of three main stages: downsampling (using a stride of 2), transformation through residual blocks, and upsampling (with a scale factor of 2) to generate the night-time image.

**Figure 4:**
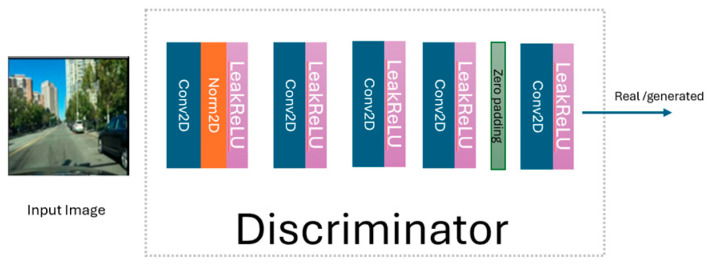
The discriminator in LBP-CycleGAN.

The generated night-time image enters the discriminator (see Figure 4) to be classified as real or generated. To improve this classification, an 8 × 8 patch GAN is used [28]. To stabilize the discriminator’s training, the LBP-CycleGAN model stores a buffer containing a mix of the last 50 generated images from the generator. This buffer helps to maintain a balance between the generator and discriminator during training. The discriminator then becomes more adept at distinguishing the generator’s latest outputs. The same procedure is used to transform night-time images into daytime images.

**Figure 5:**
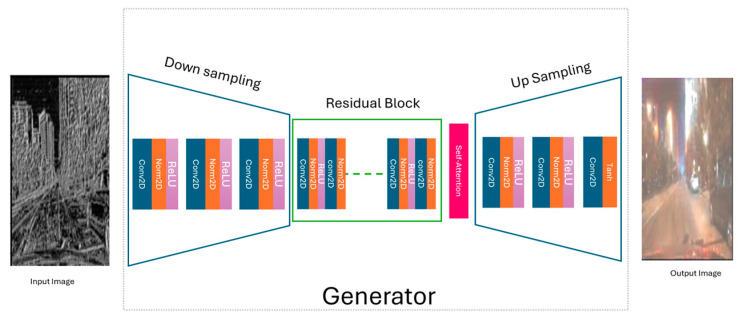
The generator in LBP-CycleGAN with self-attention.

**Figure 6:**
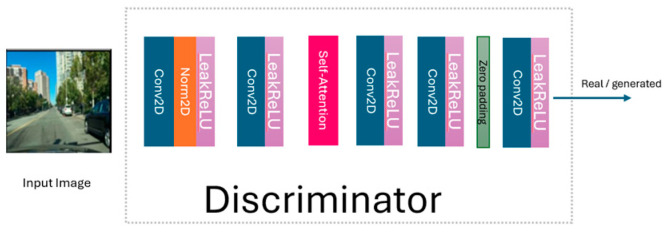
The discriminator in LBP-CycleGAN with self-attention.

We added a self-attention mechanism to the LBP-CycleGAN model to improve image generation and reduce the training time in the generator and discriminator. We then compared the model with traditional CycleGAN [1] and LBP-CycleGAN models without self-attention. We added the self-attention mechanism into the generator after the residual blocks (see Figure 5) and into the discriminator after the 2D convolution networks (see Figure 6).

Although these images travel in a CycleGAN, we compute the total loss function (total_loss) for the LBP-CycleGAN, that is, the total adversarial loss (loss_GAN), cycle consistency loss (loss_cycle), identity loss (loss_identity), and PL (percepual_loss), per the following equation:

$${total}_{loss}=loss_{GAN}+\alpha \times loss_{cycle}+\beta \times loss_{identity}+\delta \times percepual\text{\_}loss$$

where α, β, and δ are small constants used to prevent multiplication by zero. These totals help LBP-CycleGAN learn to transform images.

In our proposed method, the key novelty lies in the input representation. Unlike conventional approaches, we utilize a one-channel input Local Binary Pattern (LBP) image, as our primary focus is on night-time image generation. The motivation behind this choice is that texture transfer is more critical than color preservation in our application. Additionally, using a single-channel input significantly reduces the computational complexity and memory consumption, leading to faster training times while maintaining the essential structural details necessary for effective image translation.

### 3.4. Model Complexity and Computational Cost

To evaluate the computational efficiency of our LBP-CycleGAN model, we analyze its complexity with and without self-attention.

Without self-attention: Computational complexity remains linear in image size and is given by

$$O(N.C^{2}.H.W)$$

where N is the batch size, C is the number of channels, and H×W is the spatial resolution.

With self-attention: the complexity increases to quadratic in terms of spatial dimensions due to global pairwise interactions:

$$O(N.C.{\left(H.W\right)}^{2})$$

This increased complexity can lead to higher memory consumption and computational overhead, especially for high-resolution images.

LBP-based preprocessing: Our model incorporates Local Binary Patterns (LBPs) as a preprocessing step. Since LBP operates on local neighborhoods using pixel-wise comparisons, its computational cost is negligible compared to deep convolutional layers. As a result, LBP does not significantly affect the overall complexity of our model, making it feasible for real-time application.

By maintaining a balance between efficiency and performance, LBP-CycleGAN remains suitable for real-time applications in autonomous driving and surveillance.

## 4. Experiment and Results

This section demonstrates LBP-CycleGAN’s performance through a series of randomly generated pictures. Our LBP-CycleGAN model is implemented using the PyTorch framework (version 2.5.1) with Python 3.10. The experiments were conducted on a gaming PC equipped with an Intel Core i9 CPU, an NVIDIA GTX 4080 GPU (16 GB of VRAM), and 64 GB of RAM.

Dataset Splits:

- Training: BDD100K [29] (32,821 images for day, 24,595 images for night).
- Validation: BDD100K (5258 images for day, 3929 images for night).
- Testing: BDD100K (3656 images for day, 2736 images for night), Argoverse 2 (AV2) [30] (1052 images for day, 190 images for night), and Car Crash Dataset (CCD) [31] (6771 images for day, 729 images for night).

All images are resized to 128 × 128 for computational efficiency.

- Training Configuration:
- Batch Size: 8.
- Number of Epochs: 200.
- Optimizer: Adam (lr = 0.0001, β1 = 0.5, β2 = 0.999).

We can compare the effect of change on the model, LBP-CycleGAN with a self-attention mechanism in the generator and discriminator (Model 1), the architecture of which is illustrated in Figure 4 (generator) and Figure 5 (discriminator); LBP-CycleGAN with a self-attention mechanism in the discriminator only (Model 2), the architecture of which is illustrated in Figure 2 (generator) and Figure 5 (discriminator); and LBP-CycleGAN without a self-attention mechanism (Model 3), the architecture of which is illustrated in Figure 2 (generator) and Figure 3 (discriminator), as detailed in Table 1.

The dataset used to train the models was BDD100K, the largest driving image dataset. It has a wide range of geographical, environmental, and weather diversity, which makes it useful for training models that are not easily affected by new conditions. The dataset contains both daytime and night-time images that support our methodology.

AV2 is a collection of data that includes high-resolution imagery from professional ring cameras, a form of 360-degree camera that records images in all directions. We only used images from the front direction and divided these images according to day and night; the night-time images were obtained during twilight time only.

CCD is a publicly available dataset of dashcam videos that have been annotated with labels indicating whether an accident occurred. The dataset contains over 1000 videos from different cities and highways in China.

We used the BDD100k dataset for training and the AV2 and CCD datasets to evaluate additions to the BDD100k dataset.

We used one-channel LBP images as input for all models, except traditional CycleGAN [1], for which we used color images as input. We computed the Fréchet Inception Distance (FID) score (a lower score means a better model), which is defined as

FID=||μr−μg||2+ TrΣr+Σg−2ΣrΣg12

where the following apply:

- $\mu_{r},\;\Sigma_{r}$ are the mean and covariance of the real images’ feature embeddings;
- $\mu_{g},\;\Sigma_{g}$ are the mean and covariance of the generated images’ feature embeddings;
- $T_{r}\left(.\right)$ denotes the trace of a matrix.

And Inception Score (IS) (a higher score means a better model) is calculated as

$$IS=exp(E_{x}D_{KL}(p(y/x)||p(y)))$$

where the following apply:

- p(y/x) is the conditional label distributional from the inception model;
- $p\left(y\right)=\int_{x}(p(y/x)p(x)dx\;$ is the marginal class distribution;
- $D_{KL}$ is the Kullback–Leibler divergence between the conditional and marginal distributions.

And we compared the results. In general, the IS score focuses on the classification confidence and diversity of generated images, and the FID score provides more holistic evaluation by comparing the entire distribution of features between real and generated images.

The experiment showed that adding the self-attention mechanism increased the training time and decreased the accuracy of the models. Self-attention requires computing pairwise relationships between all spatial locations, leading to quadratic complexity. LBP features can achieve similar functionality but with significantly lower computational cost. Similar to Latent Graph Attention (LGA) [22] attention, LBP operates within a local neighborhood, using a 9-pixel connectivity pattern to extract spatial relationships efficiently. While LBP applies a fixed thresholding mechanism to encode texture patterns, LGA improves upon this by introducing learnable edge weights between locally connected nodes. Instead of computing attention over the entire image as in self-attention, LGA constructs a graph-based adjacency matrix, where each node communicates only with its eight immediate neighbors and itself. This structured local connectivity, combined with a lightweight 1 × 1 convolutional layer for learning edge weights, allows LGA to propagate information efficiently while avoiding the high computational cost of global attention mechanisms. By leveraging local dependencies rather than computing global pairwise interactions, LBP provides efficient alternatives to self-attention, making them particularly suitable for resource-limited environments such as real-time image processing and embedded systems.

Table 2 shows the experimental results for all models with three datasets: BDD100k, AV2, and CCD. The results in Table 2 indicate that the AV2 dataset in LBP-CycleGAN without a self-attention mechanism model achieves the best FID scores across all datasets. This can be attributed to the high-quality images captured using professional cameras, which provide superior clarity and detail compared to dashcam datasets.

The addition of the self-attention mechanism to the generator and discriminator in LBP-CycleGAN negatively affected the FID score. This is because the number of features in the input image affects the efficiency of self-attention [25], and in this case, only eight features were present. The LBP-CycleGAN with a self-attention mechanism in the discriminator only achieved a better FID score because the mechanism was only added to the discriminator, and the input for the discriminator is a color image. In LBP-CycleGAN without a self-attention mechanism, the results were notably improved because of the relationship with the number of features in the image. Using a self-attention mechanism typically decreases the quality of the transformed image because, as mentioned in [25], in grayscale images, self-attention struggles to effectively capture structural details due to the lack of color information, leading to weaker feature extraction. Since Local Binary Patterns (LBPs) primarily rely on texture and edge-based features, self-attention can inadvertently smooth out these critical details by distributing attention weights across similar-intensity regions, reducing the model’s ability to preserve fine-grained local structures. Additionally, self-attention tends to work better when there is a high degree of variation in the input data, which is more pronounced in color images but limited in grayscale ones. The reliance on global contextual understanding also means that self-attention may overlook small yet important texture patterns crucial for LBP-based processing. As a result, the model incorporating self-attention performed worse in terms of feature retention and recognition accuracy compared to the model without self-attention, which preserved the detailed local textures more effectively.

LBP-CycleGAN with a self-attention mechanism in the generator and discriminator and LBP-CycleGAN with a self-attention mechanism in the discriminator only had higher FID scores because of the increased model complexity and training instability that self-attention introduces, leading to slower convergence and unstable adversarial training.

LBP-CycleGAN without a self-attention mechanism achieved a better FID score than traditional CycleGAN [1] and the other two models. Using one-channel LBP images as input positively affects the transformation of daytime images to night-time images because the texture features and object edges in night-time images have a good effect on the FID score.

When comparing the IS scores, we found the opposite results: LBP-CycleGAN with a self-attention mechanism in the generator and discriminator achieved a better model score than the other models. This model contains a self-attention mechanism that learns to represent the features of the training data more effectively and produce more recognizable images that the inception model can classify confidently, even if the image does not closely resemble the real image. This had a negative effect on the FID score for the self-attention mechanism in the generator and discriminator. However, all models had similar IS scores because they all generated similarly recognizable and diverse images.

The difference between the high IS and low FID scores emerges because IS focuses more on the recognizability and diversity of the generated images, whereas FID assesses how well the total distribution of the generated images matches the distribution of real images. A high IS combined with a poor FID often suggests that the generated images are recognized and diversified but may have worse visual quality or distributional mismatch.

**Figure 7:**
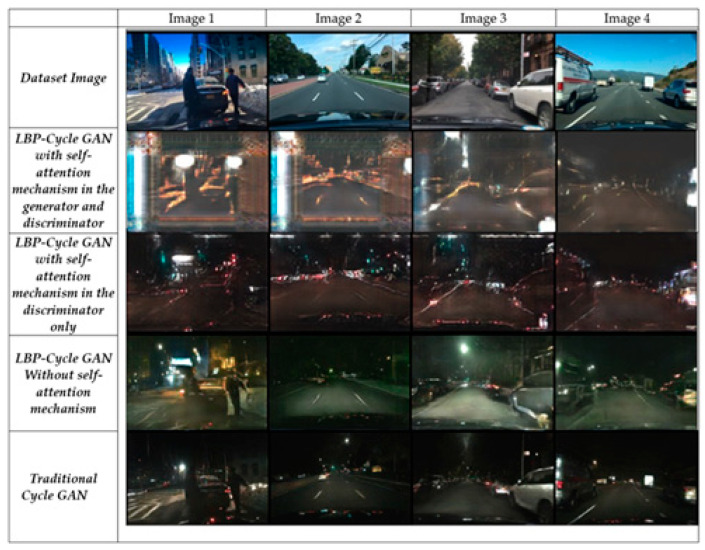
Results of the models’ image transformations using BDD dataset.

Figure 7 illustrates the three LBP-CycleGAN models and the traditional CycleGAN [1], visualizing the FIDs and ISs so that the images can be examined with the naked eye. LBP-CycleGAN without a self-attention mechanism generates sharper textures and preserves structural details more effectively.

**Figure 8:**
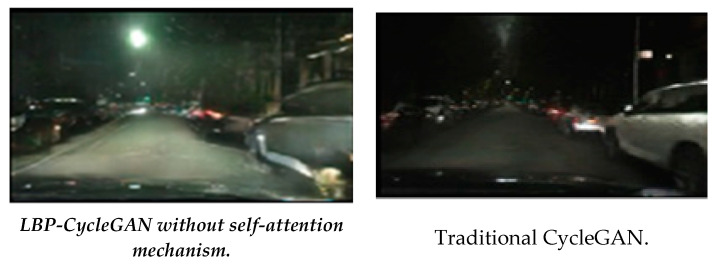
Comparison of Image 3’s transformation with LBP-CycleGAN without a self-attention mechanism and traditional CycleGAN [1].

For example, Image 3 from the LBP-CycleGAN without a self-attention mechanism provides more details of the buildings behind the car; the road surface is also more clearly marked. For this reason, this model had a smaller FID than the other models, as shown in Figure 8.

**Figure 9:**
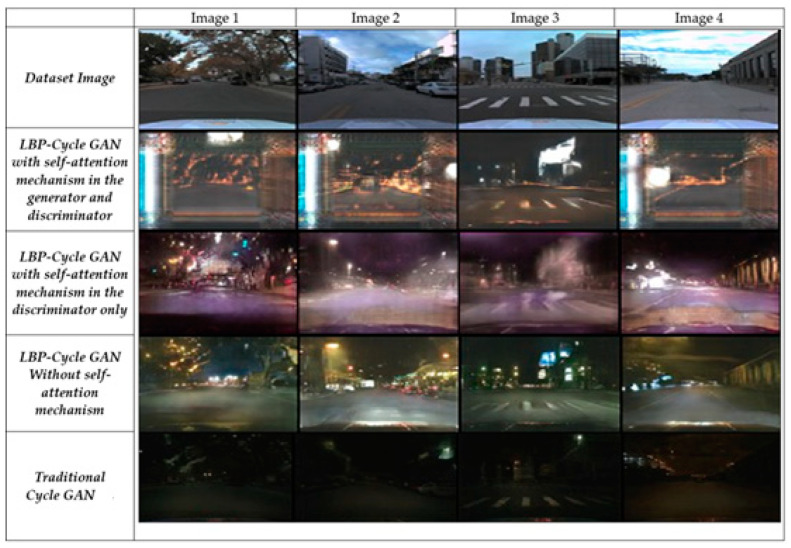
Results of the models’ image transformations using AV2 dataset.

Figure 9 illustrates the results of all models when applied to the AV2 dataset. Notably, the last row (traditional CycleGAN [1]) generates the darkest night-time images, indicating poorer performance in maintaining structural and textual details. Row 3 (LBP-CycleGAN without self-attention) produces the most realistic night-time road images, closely resembling the actual scene. This model preserves road markings, buildings, and trees with high clarity, making them distinguishable to the human eye. In contrast, LBP-CycleGAN with self-attention in the generator and discriminator) introduces more noise, reducing image quality.

**Figure 10:**
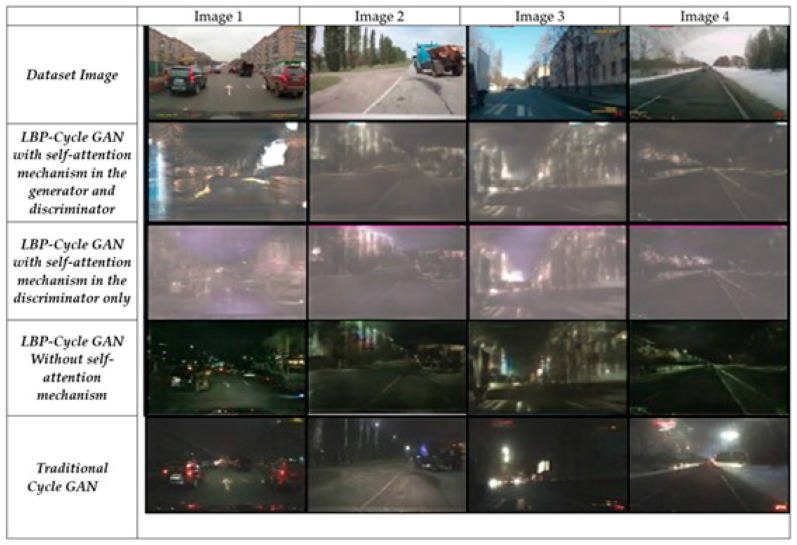
Results of the models’ image transformations using CCD dataset.

Figure 10 presents the model output on the CCD dataset, where images were captured using dashcams. Key observations include that all models effectively preserve edges and small details, but LBP-CycleGAN without self-attention enhances edge brightness, making structures more distinguishable. This model produces clearer road markings and enhanced visibility of road elements, making the output more meaningful for autonomous driving systems.

Overall, LBP-CycleGAN without self-attention achieves superior structural clarity and object preservation across different datasets, reinforcing its potential for real-world night-time vision applications in autonomous driving.

**Figure 11:**
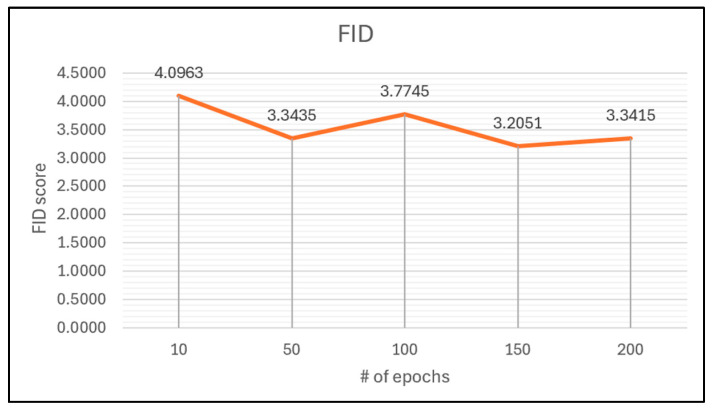
FID scores throughout the training of the LBP-CycleGAN without a self-attention mechanism.

Figure 11 illustrates the progress of the FID training of the LBP-CycleGAN without a self-attention mechanism. At 10 epochs, the FID score is 4.096; this is the highest number, meaning that it is the worst score during the training. The best score is 3.2 at 150 epochs, and at 200 epochs, the score is increased to 3.3.

**Figure 12:**
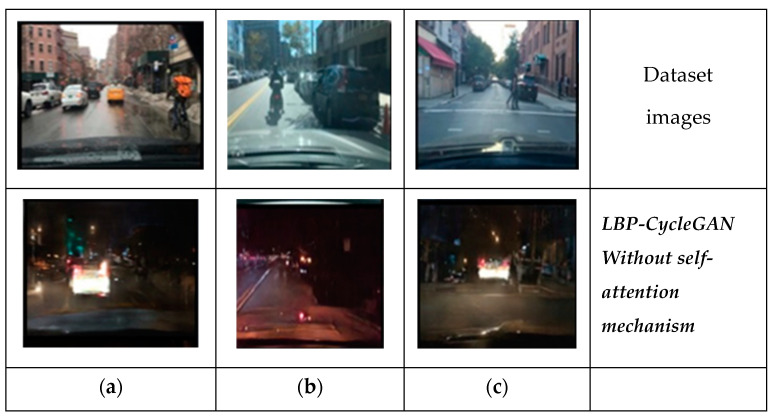
Results for LBP-CycleGAN without a self-attention mechanism compared to real images from the dataset (**a**), the bicyclist in the dataset daytime image does not appear in the generated night-time image, (**b**) the motorcycle driver in the daytime image from the dataset does not appear clearly in the generated night-time image, (**c**) the pedestrians are clear but the location of the car behind them is wrong.

However, we found problems with the LBP-CycleGAN without a self-attention mechanism. Although it had the best score, not all objects appeared in the transformed images. For example, in Figure 12a, the bicyclist in the dataset daytime image does not appear in the generated night-time image. In Figure 12b, the motorcycle driver in the daytime image from the dataset does not appear clearly in the generated night-time image. Conversely, the pedestrians are clear in Figure 12c, but the location of the car behind them is wrong.

Finally, after comparing these images, we can see that the one-channel LBP input image improved the transformation; it lightens the road using other generator input images to enhance the transformed images.

Our experimental results confirm that while diffusion models produce high-quality, globally coherent images, they require significantly higher computational resources and inference time, making them unsuitable for real-time deployment in autonomous driving. Previous studies on diffusion-based methods (DDPMs and LDMs) have demonstrated their ability to generate visually appealing images through iterative refinement [3,32]. However, these models often require extensive computational power and may struggle with preserving fine structural details such as road markings and small obstacles, which are critical for safety-critical applications. While our study does not include a direct experimental comparison with diffusion models, we reference prior works to highlight the trade-offs between image quality and efficiency. In contrast, LBP-CycleGAN without self-attention preserves clearer edges and textures, ensuring better visibility of road elements while maintaining computational efficiency across datasets, including BDD100k, AV2, and CCD. These findings suggest that while diffusion models are highly effective for photorealistic image synthesis, LBP-CycleGAN provides a more practical and efficient solution for real-time night-time vision applications.

## 5. Conclusions

In this study, we introduced LBP-CycleGAN, a novel generative model designed for night-time image translation in autonomous driving and low-light vision applications. Unlike traditional CycleGAN [1] models, which often struggle with texture degradation and structural inconsistencies, our approach leverages Local Binary Patterns (LBPs) as an input feature to enhance texture preservation while reducing computational complexity.

Through extensive experimentation, we evaluated three variations of LBP-CycleGAN:

Model 1: LBP-CycleGAN with a self-attention mechanism in the generator and discriminator.

Model 2: LBP-CycleGAN with a self-attention mechanism in the discriminator only.

Model 3: LBP-CycleGAN without a self-attention mechanism.

Our findings demonstrate that Model 3 (LBP-CycleGAN without a self-attention mechanism) outperforms the other variations, achieving the best balance between image realism, structural consistency, and computational efficiency. The results indicate that self-attention does not always enhance traditional CycleGAN [1] performance, particularly in texture-sensitive transformations. Additionally, while the LBP improves fine-texture representation, it can slightly degrade color consistency, suggesting the need for future enhancements in color-preserving feature extraction.

Moreover, when compared to diffusion models, such as Denoising Diffusion Probabilistic Models (DDPMs) [2] and Latent Diffusion Models (LDMs) [3], LBP-CycleGAN offers a more efficient and practical solution for real-time applications. While diffusion models generate highly realistic and globally consistent images, they require substantially longer inference times, making them less suitable for real-time autonomous driving. Additionally, diffusion models often over-smooth fine details, which can be a limitation in night-time driving scenarios where precise road markings and object boundaries are critical. In contrast, LBP-CycleGAN without self-attention preserves sharper textures, clearer road lines, and finer object details, making it a more reliable choice for safety-critical applications.

### Limitations and Future Work

▪ Feature Representation Limitations: While our model integrates LBP for texture enhancement, it does not yet incorporate adaptive feature fusion with deep embeddings, which could further improve texture preservation and color consistency.
▪ Limited Dataset Diversity: Our current evaluation focuses on autonomous driving datasets (BDD100K, CCD, and AV2). However, testing on indoor environments, aerial imagery, or medical imaging is necessary to assess broader applicability.
▪ Color Consistency Challenges: Some generated images exhibit chromatic distortions, which could be addressed by incorporating improved loss functions such as color consistency losses.
▪ Real-Time Processing Constraints: Although our model is computationally efficient, further optimization is required for low-latency deployment in real-world systems, especially for autonomous driving.
▪ Comparison with Advanced Models: While we have discussed diffusion models in general, a more detailed direct comparison with state-of-the-art approaches (e.g., transformers and diffusion-based models) is necessary to evaluate trade-offs in image quality, computational efficiency, and inference speed.

Future work will focus on addressing these limitations by integrating adaptive feature fusion, improving color consistency through advanced loss functions, expanding dataset evaluation, and optimizing the model for real-time applications. Additionally, we plan to conduct direct comparisons with state-of-the-art models to further assess the trade-offs between image quality, computational efficiency, and real-world applicability.

## Figures and Tables

**Table 1:** Experimental models.

Component	Model 1	Model 2	Model 3	Cycle GAN
Self-attention in generator	Y	N	N	N
Self-attention in discriminator	Y	Y	N	N
PL	Y	Y	Y	N
Identity loss	Y	Y	Y	N
Number of residual blocks	3	3	3	3

**Table 2:** Experimental results.

Model	LPB	Dataset						No. of Epochs
		BDD		AV2		CCD		
LBP-CycleGAN with self-attention mechanism in the generator and discriminator	Y	FID	5.7258	FID	8.2065	FID	5.1540	200
		IS	1.0110	IS	1.0109	IS	1.0049	
LBP-CycleGAN with self-attention mechanism in the discriminator only	Y	FID	3.63212	FID	2.943404	FID	4.3368	200
		IS	1.00026	IS	1.00066	IS	1.0	
LBP-CycleGAN without self-attention mechanism	Y	FID	3.34152	FID	2.861429	FID	4.052196	200
		IS	1.00030	IS	1.001428	IS	1.0000331	
CycleGAN	N	FID	3.77880477	FID	3.67173886	FID	4.04509544	200
		IS	1.00000560	IS	1.00000560	IS	1.0068649	

## Data Availability

The datasets used in this study are publicly available. BDD100K can be accessed at https://bair.berkeley.edu/blog/2018/05/30/bdd/ (accessed on 25 March 2025), AV2 can be accessed at: https://www.argoverse.org/av2.html (accessed on 25 March 2025), CCD can be accessed at: https://www.kaggle.com/datasets/asefjamilajwad/car-crash-dataset-ccd (accessed on 25 March 2025).
